# Correction to “Fluorescent Multiplex Immunohistochemistry (mIHC) Analyses of Local Advanced Gastric Cancer Patients After Neoadjuvant Chemotherapy Revealed the Status of Tumor‐Infiltrating Immune Cells and Predicted the Clinical Outcome”

**DOI:** 10.1002/cam4.72170

**Published:** 2026-08-06

M. Gou , Y. Gao , Y. Zhang , et al., “Fluorescent Multiplex Immunohistochemistry (mIHC) Analyses of Local Advanced Gastric Cancer Patients After Neoadjuvant Chemotherapy Revealed the Status of Tumor‐Infiltrating Immune Cells and Predicted the Clinical Outcome,” Cancer Medicine 15, no. 7 (2026): e72132, 10.1002/cam4.72132.42499012 PMC13400833

In the above mentioned article, the author Zhi Qiao has been added as co‐corresponding author.

The online version of this article has been corrected accordingly.

We apologize for this error.

